# Outcomes of hyperbolic tapering of antidepressants

**DOI:** 10.1177/20451253231171518

**Published:** 2023-05-09

**Authors:** Jim van Os, Peter C. Groot

**Affiliations:** Department Psychiatry, UMC Utrecht Brain Centre, University Medical Centre Utrecht, Postbus 85500, 3508 GA Utrecht, The Netherlands; Department of Psychiatry and Neuropsychology, Maastricht University Medical Centre+, Maastricht, The Netherlands; Department of Psychosis Studies and King’s Health Partners, Institute of Psychiatry, Psychology & Neuroscience, King’s College London, London, UK; User Research Centre Netherlands, UMC Utrecht Brain Centre, University Medical Centre Utrecht, Utrecht, The Netherlands

**Keywords:** antidepressants, dependence, drug withdrawal symptoms, patient medication knowledge, tapering

## Abstract

**Background:**

In patients attempting to discontinue their antidepressant medication, there have been no prospective studies on patterns of withdrawal as a function of the rate of antidepressant reduction during the tapering trajectory, and moderators thereof.

**Objective:**

To investigate withdrawal as a function of gradual dose reduction.

**Design:**

Prospective cohort study.

**Methods:**

The sampling frame consisted of 3956 individuals in the Netherlands who received an antidepressant tapering strip between 19 May 2019 and 22 March 2022 in routine clinical practice. Of these, 608 patients, majorly with previous unsuccessful attempts to stop, provided daily ratings of withdrawal in the context of reducing their antidepressant medications (mostly venlafaxine or paroxetine), using hyperbolic tapering strips offering daily tiny reductions in dose.

**Results:**

Withdrawal in daily-step hyperbolic tapering trajectories was limited, and inverse to the rate of taper. Female sex, younger age, presence of one or more risk factors and faster rate of reduction over shorter tapering trajectories were associated with more withdrawal and differential course over time. Thus, sex and age differences were less marked early in the course of the trajectory, whereas differences associated with risk factors and shorter trajectories tended to peak early in the trajectory. There was evidence that tapering in weekly larger steps (mean per-week dose reduction: 33.4% of previous dose), in comparison with daily tiny steps (mean per-day dose reduction: 4.5% of previous dose or 25.3% per week), was associated with more withdrawal in trajectories of 1, 2 or 3 months, particularly for paroxetine and the group of other (non-paroxetine, non-venlafaxine) antidepressants.

**Conclusion:**

Antidepressant hyperbolic tapering is associated with limited, rate-dependent withdrawal that is inverse to the rate of taper. The demonstration of multiple demographic, risk and complex temporal moderators in time series of withdrawal data indicates that antidepressant tapering in clinical practice requires a personalised process of shared decision making over the entire course of the tapering period.

## Introduction

A growing body of literature exists on the phenomenon of (persistent) antidepressant withdrawal, occasioned by the unwinding of drug-induced neurophysiological adaptation following reduction or cessation of medication.^1–5^ Practical knowledge in this area was developed initially by people with lived experience,^6–9^ but now gradually is becoming more ‘mainstream’.^10–12^ There is consensus that antidepressant withdrawal may be viewed through the lens of drug withdrawal in general, such as withdrawal associated with reduction of cessation of benzodiazepines and opiates.^6^ This view has much to offer given the solid evidence base supporting treatment of withdrawal by gradual and personal tapering of the substance in question, titrated against the degree of withdrawal discomfort. The recently described *Horowitz-Taylor method* of withdrawal represents such a common sense strategy.^9^ It remains difficult to implement for antidepressants, however, given that virtually all medications come in dosages that do not allow for flexible and personal tapering^6^ and guidelines typically do not provide practical or workable solutions.^13,14^

## Hyperbolic tapering

The *Horowitz-Taylor method* for personalised tapering of psychiatric medication recognises that tapering should be ‘hyperbolic’ to achieve a linear reduction of receptor occupancy to prevent withdrawal,^9,15^ which is otherwise more likely to occur, especially at the end of a taper when lower than registered dosages are required, which were and still are not provided by pharmaceutical companies.^6^ Hyperbolic means that the steps by which the dose is lowered are made smaller and smaller as the dose decreases.^9,15^ Hyperbolic tapering is essentially what many patients, implicitly and without using the word hyperbolic, have been advocating for many years and have tried to achieve themselves by applying do-it-yourself pharmacotherapy.^7,8,16,17^ Hyperbolic tapering has also been implicitly advocated by some professionals,^18,19^ and it was the basic idea behind the development of tapering medication in the Netherlands.^17,20^

## Hyperbolic tapering strips

In response to antidepressant users who wished to taper off their medications, the not-for-profit organisation Cinderella Therapeutics in the Netherlands oversaw the development of personal tapering strips for hyperbolic reduction of antidepressant medication in those suffering withdrawal or deemed at risk.^6,20^ A tapering strip consists of antidepressant medication, packaged in a roll or strip of small daily pouches. Each pouch is numbered and has the same or slightly lower dose than the one before it. Strips come in series covering 28 days, and patients can use one or more strips to regulate the rate of dose reduction over time in a flexible and personalised fashion. Tapering strip trajectories can take months or, if necessary, years. Dose and day information printed on each pouch allow patients to precisely record and monitor the progress of their reduction.^6,20^ Also available are stabilisation strips, which can be prescribed to continue the patient stay on a certain dose for a while, when withdrawal symptoms occur during a taper. This gives the patient time to recover before a more gradual continuation of the taper is initiated.

## Dearth of research on patterns of withdrawal over time

Attempts have been made to study antidepressant discontinuation in the context of traditional placebo-controlled RCTs; however, these endeavours have a high risk of failing.^21,22^ In contrast to the difficulties of placebo-controlled RCTs, thousands of patients in the Netherlands have contributed to a rich and growing database of retrospective cohort data derived from the widely used, patient-invented and popular method of tapering with hyperbolic tapering strips. Three retrospective cohort studies, recruiting over 2000 patients, have shown that in patients attempting antidepressant discontinuation, around 70% was able to come off their antidepressant medication with the use of hyperbolic tapering strips^23^ over a median period of 2 months, that is, using two 28-day tapering strips. It is important to note, however, that the period of 2 months often does not reflect the entire tapering trajectory as many patients, given limited reimbursement by insurers, use tapering strips for the last, most difficult part of a much longer tapering trajectory, which not infrequently may take years. While earlier studies suggest that hyperbolic tapering can be effective for the last part of a frequently much longer trajectory, very little is known about the actual course of withdrawal in such a personalised and potentially hyperbolic tapering trajectory, and moderators thereof. It has been suggested that withdrawal may take on a ‘wave-like’ form,^24^ but anecdotal evidence indicates there is extensive heterogeneity with irregular, delayed and persistent forms of withdrawal.^7^ To our knowledge, only one case study, an *n* = 1 trial of a patient using intensive monitoring technology to follow the pattern of withdrawal during hyperbolic tapering, actually attempted to chart the course of withdrawal over time.^25^

The current study presents time series data on the level of ‘controlled withdrawal’ in the context of hyperbolic reduction of antidepressant medication, using hyperbolic tapering strips, during the process of tapering. The time series data allowed us to explore the following issue: What is the course of withdrawal during the process of personalised and potentially hyperbolic tapering and is there evidence of moderation by demographic factors (age, sex), previously reported withdrawal risk factors^23,26,27^ and tapering factors such as rate of tapering as a function of shorter or longer tapering trajectories, tapering in daily small steps or weekly larger steps, and type of antidepressant medication. It is important to note that as the specific focus of this study is on what happens *during* the tapering trajectory itself, it cannot inform about what happens with patients *after* they have tapered completely. The latter issue was investigated by us before and has been reported elsewhere.^23,26,27^

## Methods

### Sample and data collection

Patients whose doctors prescribe a tapering strip for antidepressant medication routinely receive a form, together with the tapering medication with the primary aim to help them, and the prescribing clinician, to assess the course of withdrawal symptoms over time. This idea was born from lived experience, where it was noted that the process of shared decision making around individual tapering trajectories is best guided by sufficient monitoring data.^6^ A single monitoring form covers 28 days, and patients are instructed to rate, at the end of each day, the level of suffering due to withdrawal on a scale from 1 to 7 (1 = not at all, 2 = very little, 3 = a little, 4 = some, 5 = bearable, 6 = a lot, 7 = very much). There was no overlap between the current sample and three previous samples we reported on previously.^23,26,27^ In principle, it was possible for patients to taper hyperbolically from beginning to end. In practice, however, it was possible to flexibly adapt a tapering schedule when this was deemed necessary or desirable, by prescribing (1) a more gradual tapering strip(s), or prescribing (2) stabilisation strip(s) first. In fact, the reason to ask patients to fill in a self-monitoring form during the tapering trajectory was to make these adjustments practically possible, in order to prevent withdrawal during the tapering trajectory. As a result, not all, and probably not even most, participants in the sample followed tapering schedules which were hyperbolic from beginning to end.

Of 3956 individuals who received an antidepressant tapering strip between 19 May 2019 and 22 March 2022, 655 (17%) returned one or more forms monitoring their trajectory. Excluded were 47 individuals who had used only or mostly stabilisation strips. These 47 also included 16 individuals with extreme tapering trajectories longer than 5 months who had an unusual high rate of using stabilisation strips (38% *versus* 6% in remainder). This left 608 individuals for analysis (16% of total sample), who had a total of 32,368 observations over the course of their tapering trajectories. Of the 608 individuals, 288 (47%) had a tapering trajectory of 28 days, 170 (28%) of 56 days, 88 (15%) of 84 days, 41 (7%) of 112 days and 21 (4%) of 140 days. Two individuals had missing values on type of tapering (daily tiny steps or weekly larger steps, see below).

Sample selection was such that all patients who were at any point in their tapering trajectory during the sampling selection period were selected. Thus, some patients in the sample would have had one 28-day tapering strip over the sample selection period, whereas their second and later tapering strips fell outside the sample selection period and thus would not have been included in the analysis. The sample thus consisted of some patients whose entire tapering trajectory was included, and some patients whose tapering trajectory was only partially included. For each patient, the difference between the dose at the beginning and at the end of the tapering trajectory over the sampling selection period was expressed as percentage dose reduction or (1 − (end dose / initial dose)) × 100%, with dose expressed as units daily defined dose (ddd). For example, a patient with an initial dose of 0.80 ddd and an end dose of 0.20 ddd would have a dose reduction of 75%. Given the time-based cutoff in the sampling selection period, the percentage dose reduction was higher for individuals with longer tapering trajectories (percentage dose reduction 28 days: 63.0%; 56 days: 84.0%; 84 days: 92.5%; 112 days: 94.4%; 140 days: 87.5%).

Also used in the analyses was the average rate of weekly dose reduction, expressed as percentage of initial dose. For example, if an individual had reduced the those from 8 to 6 mg paroxetine in week 4 of the tapering trajectory, having started the tapering trajectory at an initial dose of 20 mg, this would represent an 10% [((8 − 6)/20) × 100%] dose reduction.

### Hyperbolic tapering in tiny daily steps *versus* weekly large steps

A small number of patients in the sample (8.6%) were not prescribed a hyperbolic tapering strip with daily tiny steps but instead followed a schedule of weekly larger dose reductions. The weekly steps were proposed by a policy group in 2018^28^ but are rarely prescribed by doctors as this type of tapering is thought to be associated with higher levels of withdrawal. As weekly steps, according to the policy group, are to be prescribed for five antidepressants (venlafaxine, paroxetine, citalopram, fluvoxamine, sertraline), samples were group-matched for antidepressant used. This resulted in 456 subjects treated with a daily tiny-step tapering strip and 52 with a weekly large-step tapering strips in these five antidepressant medications. Of the 52 in the large-step tapering group, 18 had a trajectory length of 28 days, 18 of 56 days, 7 of 84 days, 8 of 112 days and 1 of 140 days. Given the presence of only a single individual in the longest tapering trajectory, analyses comparing weekly large-step and daily tiny-step tapering were restricted to the first four trajectories, resulting in 51 individuals in the weekly large-step tapering group and 440 individuals in the daily tiny-step tapering group, yielding a total sampling frame for this subanalysis of *n* = 491.

### Analyses

Panel data were modelled taking into account intra-personal clustering of observations using multilevel random regression in Stata (version 16). The full model included level of withdrawal as response variable and the following explanatory variables: age (three groups 1 = ⩽35 years, 2 = 36–60, 3 = >60), sex (0 = male, 1 = female), type of antidepressant (0 = other antidepressant, 1 = paroxetine, 2 = venlafaxine), number of risk factors, time (in weeks), total length of tapering trajectory (1 = 28 days, 2 = 56 days, 3 = 84 days, 4 = 112 days, 5 = 140 days) and antidepressant dose (in units of daily defined dose of the antidepressant in question, divided by their quintiles so as to create quintile groups, quintile group 1 reflecting the lowest, and quintile group 5 the highest dose). Explanatory variables were entered as factored variables to account for non-linear effects, with additional calculation of summary association linear trend for continuous variable. The independent variable time was expressed in weekly units, referring to the number of weeks that patients were tapering their medication over the specified period of investigation (minimum 1, maximum 20).

**Figure 1.**
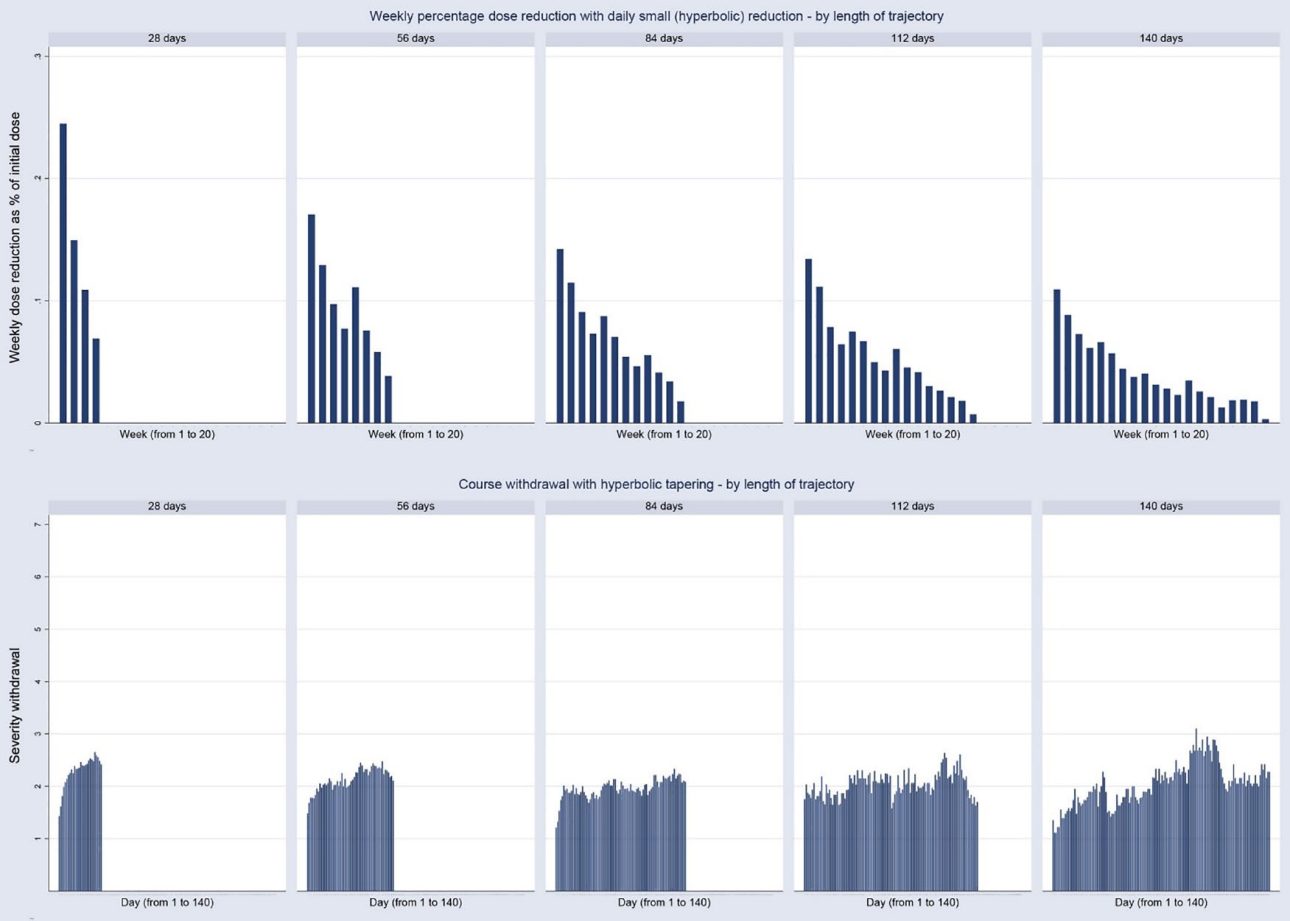
Marginsplot of interaction between time and sex in the model of standardised withdrawal ratings.

**Figure 2.**
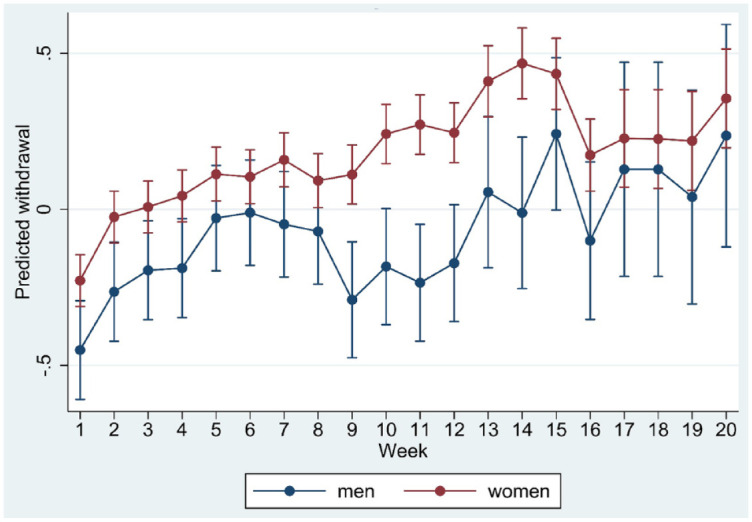
Marginsplot of interaction between time and age group in the model of standardised withdrawal ratings.

**Figure 3.**
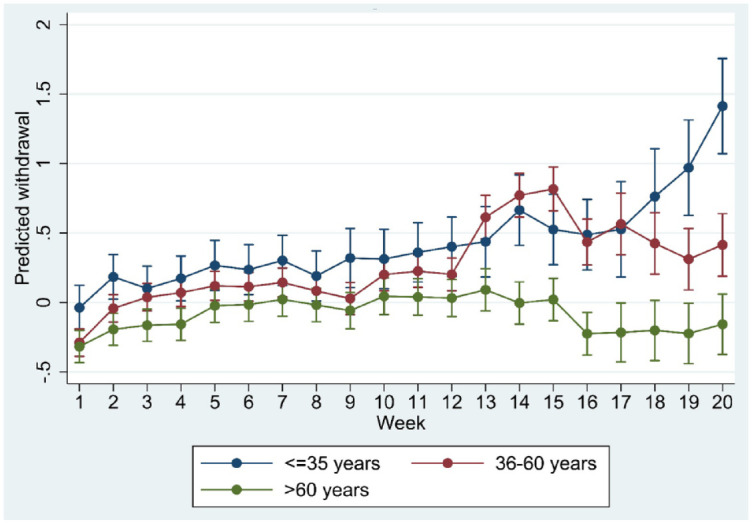
Marginsplot of interaction between time and risk load in the model of standardised withdrawal ratings.

**Figure 4.**
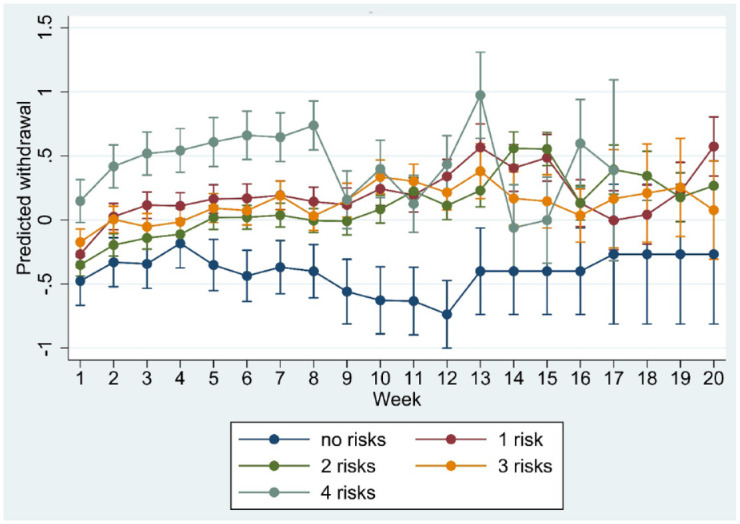
Marginsplot of interaction between time and length of tapering trajectory in the model of standardised withdrawal ratings.

We examined statistical models fitting interactions between these moderators and time, with time entered as a factored variable, in order to allow for non-linear effects over time, creating 19 dummy variables for week with week 1 as reference category. The test for interaction was a test for the hypothesis of the resulting interaction terms simultaneously not deviating from zero. To visualise and aid interpretation of interaction models, the Stata *marginsplot* routine was used to calculate the adjusted predicted values in the various combinations of the interacted variables and draw graphs of these (Figures 1–4).^29^ The response variable of level of withdrawal was standardised (with mean zero and unit standard deviation) for ease of interpretation of reported regression coefficients (standard deviation change in response variable with one unit change in explanatory variable). Interaction models with a specific moderator (e.g. sex) were adjusted for the other moderators (age, risk load, medication type, starting dose).

## Results

### Sample attrition

Responders (*n* = 608) and non-responders (*n* = 3301) were very similar in terms of demographics, type of medication, risk factors and number of tapering strips (Table 1). The majority was female, mean age was around 50, the most common antidepressants used were venlafaxine and paroxetine and the most common risk factors were unsuccessful previous attempts, anticipation of withdrawal and use of antidepressant medication longer than 2 years.

**Table 1.** Descriptive statistics as a function of sample attrition.

Sample	Percentage or mean	SD	*n*
Responders			
Mean age	52.8	13.8	608
Female sex	80.6		608
Mean number of strips	1.8	1.1	608
Used Venlafaxine	41.3		608
Used paroxetine	23.7		608
Risk factors			
Fear withdrawal^a^	40.0		608
Previous unsuccessful attempt	52.5		608
Possible relapse	6.7		608
Slow metaboliser^b^	1.7		608
High dose^c^	3.1		608
Problems when starting	2.7		608
Problems when missing dose	8.1		608
Antidepressant use > 2 years	81.1		608
No risk factors	6.9		608
Mean number of risk factors	1.9	1.0	608
Non-responders			
Mean age	46.5	15.4	3301
Female sex	72.1		3301
Mean number of strips	2.2	1.6	3301
Used venlafaxine	37.0		3301
Used paroxetine	18.9		3301
Risk factors			
Fear withdrawal^a^	35.0		3301
Previous unsuccessful attempt	44.9		3301
Possible relapse	5.8		3301
Slow metaboliser^b^	1.2		3301
High dose^c^	2.4		3301
Problems when starting	4.2		3301
Problems when missing dose	8.8		3301
Use longer than 2 years	71.2		3221
No risk factors	14.4		3301
Mean number of risk factors	1.7	1.1	3301

a Fear of starting with medication reduction because of anticipated withdrawal.

b Patient has undergone pharmacogenetic test indicating slow/intermediate metaboliser status for current medication.

c Dose above maximum dose as recommended in the Dutch standard (Farmacotherapeutisch Kompas).

#### Sample characteristics and withdrawal over time in hyperbolic tapering

Sample characteristics are displayed in Table 2, stratified by type of tapering.

**Table 2.** Sample characteristics, by type of tapering.

	Variable	Percentage or mean	SD	*n*
Daily tiny-step tapering	Starting dose^a^	0.73	0.49	554
	Total dose reduction (as % of starting dose)	82.2	23.2	554
	Per-day dose reduction (as % of previous dose)	4.5	8.7	554
	Used venlafaxine	40.0		554
	Used paroxetine	24.1		554
	Age	53.9	13.8	554
	Percentage women	81.3		554
	Risk factors			
	Fear withdrawal	38.4		554
	Previous unsuccessful attempt	55.0		554
	Possible relapse	7.4		554
	Slow metaboliser	1.8		554
	High dose	3.7		554
	Problems when starting	3.2		554
	Problems when missing dose	6.9		554
	Antidepressant use > 2 years	82.1		554
	No risk factors	6.3		554
	Number of risk factors	2.0	1.0	554
	Length tapering trajectory in days	70.1	35.1	554
	Used stabilisation strip	6.2		554
Weekly large-step tapering	Starting dose^a^	0.45	0.44	52
	Total dose reduction (as % of starting dose)	85.8	16.3	50
	Per-week dose reduction (as % of previous dose)	33.4	17.3	50
	Used venlafaxine	51.8		52
	Used paroxetine	23.2		52
	Age	55.1	13.1	52
	Percentage women	84.8		52
	Risk factors			
	Fear withdrawal	43.8		52
	Previous unsuccessful attempt	45.5		52
	Possible relapse	0.0		52
	Slow metaboliser	3.6		52
	High dose	0.0		52
	Problems when starting	12.5		52
	Problems when missing dose	7.1		52
	Antidepressant use > 2 years	68.8		52
	No risk factors	9.8		52
	Number of risk factors	1.8	1.0	52
	Length tapering trajectory in days	76.5	32.6	52
	Used stabilisation strip	18.8		52

a Expressed in units of daily defined dose.

In the group with hyperbolic tapering in daily tiny steps (*n* = 554; i.e. excluding 52 with reduction with weekly steps and 2 with missing values on type of tapering), the distribution of antidepressant medications is shown in Table 3. Mean level of withdrawal for values of moderator variables is displayed in Table 4, suggestive of higher levels in women, younger people, lower initial dose, the shortest tapering trajectory, need for a stabilisation strip and use of paroxetine and venlafaxine.

**Table 3.** Distribution of antidepressant medication in those with tiny-step tapering.

Antidepressant	Percentage	Frequency
Amitriptyline	4.6	23
Bupropion	3.2	18
Citalopram	9.4	55
Clomipramine	2.7	11
Escitalopram	0.2	1
Fluoxetine	3.4	20
Fluvoxamine	1.9	11
Mirtazapine	4.1	20
Nortriptyline	0.4	3
Paroxetine	24.1	134
Sertraline	5.8	34
Tranylcypromine	0.2	2
Venlafaxine	40.0	222
Total	100.0	554

**Table 4.** Mean level of withdrawal for values of moderator variables in 554 individuals using daily tapering strips.

Age	Mean	SD	*n*
⩽35 years	2.2	1.3	75
36–60 years	2.2	1.3	288
>60 years	2.0	1.2	191
Number of risk factors			
0	1.7	1.3	35
1	2.1	1.4	134
2	2.1	1.2	240
3	2.1	1.2	110
4	2.3	1.3	35
Initial dose^a^			
1	2.3	1.6	103
2	2.2	1.5	92
3	2.2	1.4	128
4	2.1	1.4	120
5	1.8	1.1	111
Sex			
Men	1.8	1.0	109
Women	2.2	1.3	445
Length of tapering trajectory in days			
28	2.3	1.4	270
56	2.1	1.2	150
84	2.0	0.9	81
112	2.0	1.3	33
140	2.1	0.8	20
Use of any stabilisation strip			
No	2.1	1.3	535
Yes	2.5	1.0	19
Medication type			
Other	2.0	1.2	198
Paroxetine	2.3	1.1	134
Venlafaxine	2.1	1.4	222

a Expressed as units of daily defined dose and divided by its quintiles, to create quintile groups.

In the group of 554, the proportion of patients with previous unsuccessful attempts at discontinuation grew progressively higher in those with longer tapering trajectories (28 days: 50%; 56 days: 54%; 84 days: 59%; 112 days: 61% and 140 days: 70%). Shorter tapers meant faster dose reductions. Thus, the rate of weekly dose reduction, expressed as percentage of the dose of the week before, was highest in the shortest tapering trajectory and lowest in the longest tapering trajectory, with linear reduction over length of tapering trajectory (28 days: 29.7% per week, 56 days: 25.8%; 84 days: 23.8%; 112 days: 23.6% and 140 days: 19.2%).

**Figure 5.**
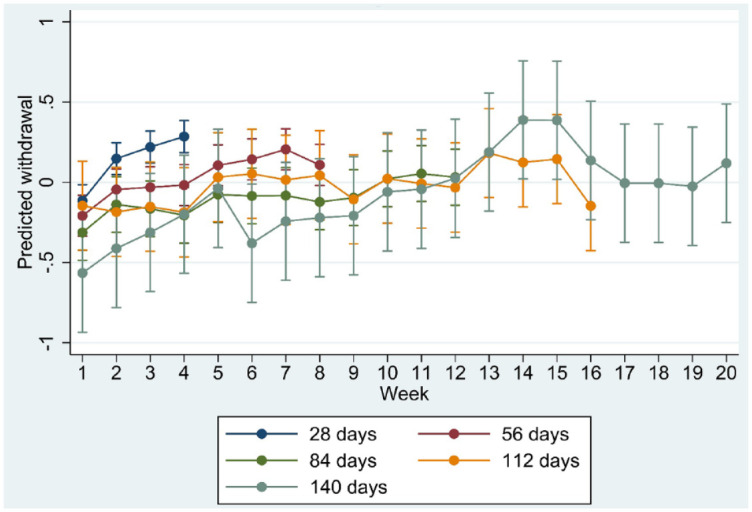
Course of daily withdrawal ratings (below) during hyperbolic antidepressant tapering (above), shown as weekly dose reduction expressed as percentage of initial dose, stratified by length of tapering trajectory.

Figure 5 shows the rate of weekly dose reduction, expressed as percentage of initial dose, in relation to daily withdrawal for each of the five tapering trajectories (28 days, 56 days, 84 days, 112 days and 140 days). Withdrawal reactions were limited, with withdrawal scores varying between ‘2’ (very little) and ‘3’ (a little). For the shortest trajectory of 28 days, a relatively fast rate of hyperbolic tapering was accompanied by a mirror image of withdrawal, with a steeper initial increase in withdrawal in the shortest trajectory compared with the longer trajectories. Longer trajectories displayed a lower rate of weekly reduction and a lower level of initial withdrawal.

### Regression analysis of withdrawal over time in hyperbolic tapering with daily tiny steps

Adjusted regression analysis mostly confirmed the impression of the graphical analysis, with evidence for stronger withdrawal in women and younger people, as well as in those with more risk factors and a shorter tapering trajectory associated with higher rate of weekly dose reduction. Use of stabilisation strip and initial starting dose, after adjustment for the other explanatory variables, were not associated with withdrawal. Venlafaxine and paroxetine were directionally – but statistically inconclusively – associated with more withdrawal (Table 5).

**Table 5.** Regression analysis of explanatory variables in multilevel regression model of daily standardised withdrawal scores.

	*b* ^a^	*p* value	95% CI low	95% CI high
Number of risk factors				
0^b^				
1	0.59	0.000	0.44	0.75
2	0.47	0.000	0.31	0.64
3	0.55	0.000	0.37	0.72
4	0.97	0.000	0.76	1.18
Linear trend^c^	0.11	0.000	0.07	0.15
Age group				
⩽35^b^				
36–60	−0.16	0.040	−0.32	−0.01
>60	−0.32	0.001	−0.51	−0.14
Linear trend^c^	−0.15	0.004	−0.24	−0.05
Female sex	0.24	0.003	0.08	0.41
Medication				
Other^b^				
Paroxetine	0.12	0.183	−0.05	0.29
Venlafaxine	0.09	0.339	−0.10	0.28
Starting dose^d^				
1^b^				
2	0.01	0.944	−0.21	0.23
3	−0.18	0.123	−0.40	0.05
4	−0.08	0.468	−0.30	0.14
5	−0.06	0.586	−0.27	0.15
Linear trend^c^	−0.03	0.279	−0.07	0.02
Any stabilisation strip	0.19	0.299	−0.17	0.56
Week				
1^b^				
2	0.20	0.000	0.17	0.23
3	0.24	0.000	0.21	0.27
4	0.27	0.000	0.24	0.30
5	0.36	0.000	0.32	0.40
6	0.35	0.000	0.31	0.39
7	0.39	0.000	0.35	0.43
8	0.33	0.000	0.29	0.37
9	0.30	0.000	0.25	0.35
10	0.43	0.000	0.37	0.48
11	0.44	0.000	0.39	0.50
12	0.43	0.000	0.38	0.49
13	0.61	0.000	0.53	0.69
14	0.65	0.000	0.57	0.73
15	0.66	0.000	0.58	0.74
16	0.39	0.000	0.31	0.47
17	0.47	0.000	0.34	0.60
18	0.47	0.000	0.34	0.60
19	0.45	0.000	0.32	0.58
20	0.59	0.000	0.46	0.72
Linear trend^c^	0.031	0.000	0.028	0.033
Length tapering trajectory in days				
1 (28 days)^b^				
2 (56 days)	−0.19	0.015	−0.35	−0.04
3 (84 days)	−0.36	0.000	−0.55	−0.17
4 (112 days)	−0.35	0.015	−0.64	−0.07
5 (140 days)	−0.43	0.022	−0.80	−0.06
Linear trend^c^	−0.13	0.000	−0.19	−0.07

CI, confidence interval.

a Regression coefficient expressing standard unit change in withdrawal with one unit change in explanatory variable. For example, for number of risk factors: compared with a person with no risk factors, a person with one risk factor has an increase in 0.59 standard deviation in withdrawal score. Overall, over five levels of risk, the mean SD increase in withdrawal with each increase in risk is 0.11.

b Reference category.

c Summary change in response variable with one unit change in explanatory variable.

d Expressed in units of daily defined dose.

Age, sex, trajectory length and risk load showed significant interaction with time in the regression model (all *p* < 0.001), suggesting differences in the course of withdrawal as a function of these moderators. Marginplots indicated that sex differences in withdrawal were most marked and statistically significant (non-overlapping confidence intervals) between week 8 and week 15 (Figure 1) whereas age differences appeared more pronounced after week 12 (Figure 2). Differences in risk load (Figure 3) and trajectory length (Figure 4) were more pronounced in the first 8 weeks.

#### Tapering with daily tiny steps *versus* weekly larger steps

The sampling frame for this comparison, as detailed above, was 456 patients in the hyperbolic group and 52 in the weekly group. The rate of dose reduction, expressed as percentage of previous dose, was 4.5% per-step in the daily tiny-step trajectory (or 25.3% per week), and 33.4% per-step in the weekly large-step trajectory.

**Figure 6.**
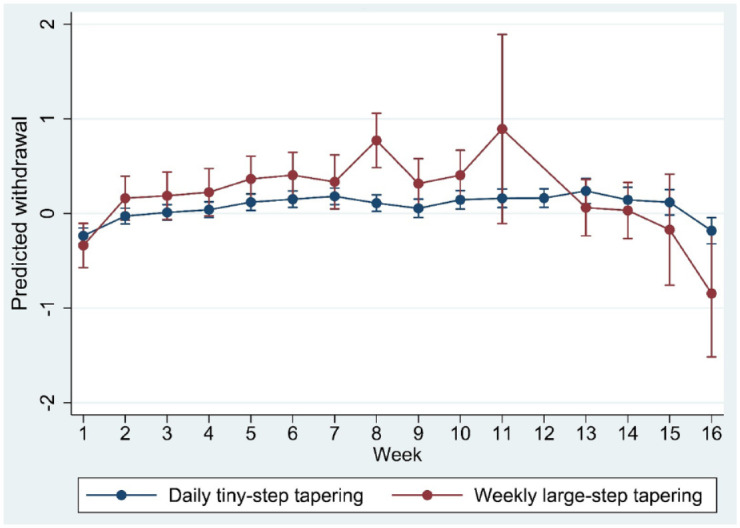
Marginsplot of interaction between time and type of tapering (daily tiny steps *versus* weekly large steps) in the model of standardised withdrawal ratings.

**Figure 7.**
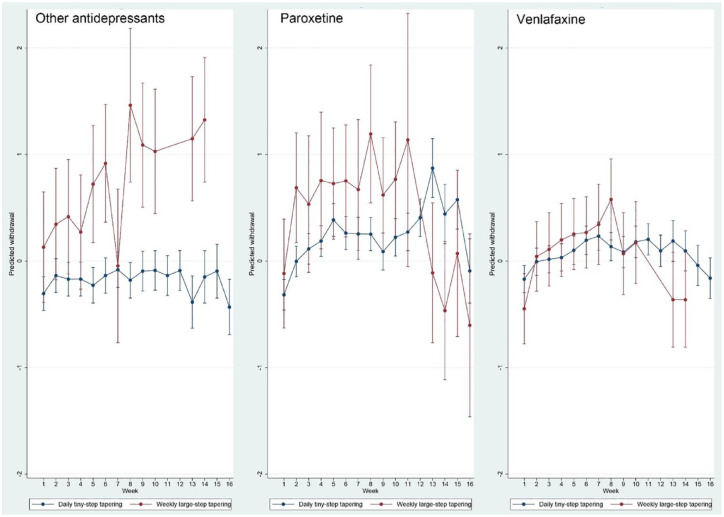
Marginsplot of interaction between time and type of tapering (daily tiny steps *versus* weekly large steps) in the model of standardised withdrawal ratings, by medication type.

Adding type of tapering to the full regression model (with age, sex, risk load, week, type of medication, length of tapering trajectory, starting dose and use of any stabilisation strips) revealed a directionally but statistically inconclusive association indicating more withdrawal in the weekly condition (*b* = 0.14, 95% CI: −0.10 to 0.38, *p* = 0.247) and a significant interaction with time (*p* < 0.001). The marginsplot showed higher withdrawal in the weekly group, with the exception of month 4 (Figure 6). Further explorative analysis by type of medication revealed marginplots with strongest evidence for interaction in the other antidepressant group, followed by paroxetine, in the first 3 months, with less evidence for interaction in the venlafaxine group (Figure 7). The suggestive tiny-step tapering main effect and the interaction remained the same after adding rate of dose reduction to the model (tiny-step tapering main effect: *b* = 0.14, *p* = 0.27; interaction *p* < 0.001), indicating that the difference in withdrawal between the tiny-step and the large-step tapering strategy was not reducible to their difference in the per-step size of dose reduction.

## Discussion

### Findings

In this report, the pattern of withdrawal following hyperbolic tapering was described in detail for a large sample of patients wishing to discontinue their antidepressant medication. Withdrawal in hyperbolic tapering in trajectories of daily tiny steps was limited (withdrawal scores varying between ‘very little’ and ‘a little’) and rate-dependent, taking the form of an approximate mirror-image of the rate of dose reduction. Female sex, younger age and presence of one or more risk factors, in addition to faster rate of reduction in shorter tapering trajectories, were associated with more withdrawal and differential course over time, indicative of non-linearity.

### Relevance for clinical practice

National guidelines on tapering of antidepressant and other psychotropic medications, if they exist at all, typically are based on ‘expert opinion’, excluding user knowledge and lived experience, and not very helpful when it comes to the actual process of tapering the medication.^1,6,13^ Cohort research, focusing on widely used and accepted ways of tapering, developed by users with lived experience, may represent a productive way to learn about the process of tapering and which factors moderate withdrawal over time. The current study appeared to be a case in point in that we established several important findings with relevance for clinical practice.

First, the study provided evidence that the mean level of withdrawal in hyperbolic tapering, with trajectories of daily tiny steps, was limited (withdrawal scores varying between ‘very little’ and ‘a little’) and that withdrawal can be expected to take the form of an approximate mirror-image of dose reduction. An important difference with previous work is that ratings in the current study were daily and prospective whereas in other work they were rated in retrospect.^23,26,27^

Second, the rate antidepressant dose reduction was associated with the level of withdrawal. The rate of weekly dose reduction was highest in the group who tapered over 28 days, and in this group a rapid rise in withdrawal was observed, mirroring the hyperbolic reduction in dose. Similarly, the rate of weekly dose reduction was progressively lower in the longer tapering trajectories and was associated with a linear trend in lower withdrawal, independent of other factors. This means that guidelines should not insist on relatively short, 28-day trajectories and provide the possibility for much longer trajectories in a process of shared decision making. Indeed, it is quite clear from this analysis that there is substantial variation in the length of the tapering trajectory that the process of shared decision making produces. There can be no prescribed trajectory length that will be valid for all. Rather, the process of shared decision making and monitoring over the course of the tapering trajectory should drive the choice for how long it will take.

Third, the evidence that rate of reduction is associated with the level of withdrawal was supported by the comparison between tapering in daily tiny steps (on average 4.5% per-daily step dose reduction – or 25.3% per week) and tapering in weekly large steps (on average 33.4% per-step dose reduction). The on-average 33.4% per-step reduction was associated with a differential course over time with evidence for more severe withdrawal in trajectories of 1, 2 or 3 months, particularly for paroxetine and the group of other antidepressants. In the regression model, the difference in withdrawal between daily tiny steps and weekly large steps was not explained by small differences in the per-step size of the weekly dose reduction.

The findings in points 2 and 3 above are compatible with the neurobiology of tapering as proposed by Horowitz and Taylor.^30^ When a psychiatric drug is administered, physiological adaptations occur, and a new homeostatic set-point is established. Abrupt discontinuation of the drug leads to withdrawal symptoms, with their duration determined by the time needed for the adaptations to resolve. These symptoms may worsen or peak even after the drug is eliminated. Tapering, or step-wise reduction of the drug, causes withdrawal symptoms at each step, but with lesser intensity than abrupt discontinuation as a new, lower homeostatic set-point is established before further dose reductions. Drugs with longer half-lives may lessen withdrawal symptoms by minimising the difference between the shifting set-point and plasma levels. Also, according to the model, a more gradual step-wise reduction further lowers the risk of withdrawal symptoms.

Fourth, paroxetine was the third, and venlafaxine the fifth most commonly prescribed antidepressant in the Netherlands in 2019 (source: vektis.nl). The fact that they were by far the most common antidepressants that tapering strips were requested for in this and previous surveys^23,26,27^ suggests, given over-representation in the study group relative to overall prescription rates, that use of these medications is associated with greater risk of withdrawal, as widely recognised (e.g. https://www.rcpsych.ac.uk/mental-health/treatments-and-wellbeing/stopping-antidepressants).

Fifth, this study, for the first time, was able to show the effect of moderators on the course of withdrawal over time. The influence of moderators may point to underlying differences in the degree of neurophysiological adaptation after antidepressant exposure and/or differences in emotional expression following the unwinding of neurophysiological adaptation. Thus, sex and age differences were less apparent early in the course of the tapering trajectory, whereas differences associated with risk factors and shorter trajectories tended to peak early in the trajectory. These suggestions of non-linearity require replication and may point to differences in underlying biological mechanisms. The clinical relevance of these moderators, and associated non-linearity, is discussed in the section below.

Finally, the use of stabilisation strips was also associated with more withdrawal, which likely reflects reverse causality in that individuals experiencing more withdrawal are more likely to require a stabilisation strip, temporarily halting the reduction in dosage, in a process of shared decision making. This suggests that stabilisation is a useful tool in the process of shared decision making guiding the process of tapering psychotropic medication.

### How may risk factors inform clinical practice?

While traditionally much emphasis has been put on risk factors for outcomes in mental health to inform clinical practice, in the sense of using group-based differences to develop an algorithm guiding clinical decision making, a more epidemiologically informed view calls for a more cautious approach. For example, suicide risk assessment procedures are commonly used in clinical practice under the assumption that they predict future behaviour and can be used as a means of allocating treatment. Established risk factors in mental health care, however, almost invariably lack sensitivity and specificity and therefore should not be used for the purpose of algorithm-based clinical decision making. Rather, low sensitivity and low specificity provide a scientific basis for the suggestion that clinical management should always be personalised and collaboratively developed with patients and their families and carers.^31^ In the domain of antidepressant tapering, several national guidelines erroneously suggest that presence of risk factors can be used as an algorithm-based decision tool to select specific treatment strategies. For example, the Dutch multidisciplinary guideline for antidepressant tapering prescribes that absence of risk factors is sufficiently predictive of absence of withdrawal for clinical management in these patients to be different than for patients with risk factors.^32^ However, given invariably weak sensitivity and specificity of risk factors, use of these in guidelines to stratify clinical management puts patients at risk of adverse outcomes. In the current study, we examined to what degree tapering outcomes are influenced by cumulative risk, expressed as a sensitive measure of risk load. This showed that there was an influence of risk load; however, the major distinction was between any risk and no risk and the effect size was typical, that is, not strong. In addition, given the fact that only 6% of patients had *no* risk factor, the conclusion is that *a priori* the role of risk factors for algorithm-based clinical decision making is limited. The findings therefore are clinically relevant as they point to multiple complex underlying sources of heterogeneity that cannot be captured in algorithm-based rules for clinical decision making, and therefore should be addressed by offering each patient a careful and highly personal process of shared decision making that continues during the entire course of the tapering trajectory.

### Methodological issues

As the study is observational, caution is required in assuming causality and its direction. Thus, the association between stabilisation strips and withdrawal likely represents reverse causality, as discussed earlier. Reverse causality may also play a role in explaining other associations. On the other hand, the association between rate of antidepressant dose reduction and withdrawal, representing the main finding of this study, likely is causal.

We cannot assume the sample is representative for the entire population of people who want to taper their antidepressant medication, even though (1) attrition analysis suggested a degree of generalisability as responders and non-responders were very similar in terms of demographics, type of medication, risk factors and number of tapering strips and (2) a sample of 17% percent of all patients who had been prescribed a tapering strip over a specific period in the entire country may be considered a fair sampling frame. First, there was extensive attrition, which may limit generalisability. Second, a degree of socioeconomic selection is likely as insurers in the Netherlands typically reimburse only 1–3 months of use of tapering strips, whereas tapering antidepressant medication in real life may take years. This also may affect generalisability.

For the interpretation of the results of this study, it is important to note that the length of a tapering trajectory indicated the time, during the specific sampling period, a patient tapered using (1) tapering strip(s) and or (2) stabilisation strip(s) and not necessarily the total time a patient used to taper completely. In other words, trajectories presented here likely include many partial tapering trajectories. Patients are limited in their options to taper hyperbolically from beginning to end, because health insurers in the Netherlands mostly reimburse 1–3 months of use. Tapering trajectories not infrequently take years; therefore, patients often select the final, most difficult part of the trajectory to use tapering strips. Only patients who can pay for tapering strips for longer periods have the option to taper hyperbolically from beginning to end over a trajectory that may take years. In this sample, most patients would likely not have done this and would have also tapered using available dosages that pharmacies can readily provide, especially at the start of a tapering trajectory when the person is still using higher dosages that are routinely available.

In the groups with the longer tapering trajectories of 56–140 days, initial doses were reduced by 84–94%. It cannot be said, however, that their tapering trajectories took between 2 and 5 months because this implies they came off the medication. The theory of receptor occupancy, indicating that the last 10–15% of medication can take longer to come off than the first 85%,^9^ implies that safe tapering may take much longer than the 2–5 months described.

A strength of the study is the focus on a novel and widely used approach that was developed by users with lived experience of withdrawal. Other strengths are large sample size, prospective daily ratings of withdrawal, national sampling frame, absence of extensive exclusion criteria and protocol requirements and a naturalistic design allowing for individual flexibility in rate and timing of tapering.

The indication for antidepressant use was not known. The question, however, is to what degree this would be a concern given that it is unlikely that tapering and withdrawal would vary qualitatively or quantitatively as a function of antidepressant indication. Furthermore, research indicates that in the Netherlands, 75% of antidepressant indications are for depression, anxiety and other mental disorders and only 25% for non-mental indications, and that this 3:1 ratio increases further in the population with persistent use, as the majority in our sample.^33^

Results on the comparison between weekly large steps and daily tiny steps were imprecise due to the small number of patients in the large-step tapering group. Replication with a larger sample would be desirable but is, given the results, unlikely to occur as doctors already rarely prescribe the weekly steps and are likely to do so even less in the future.

This study represents an epidemiological survey which, as all studies, comes with advantages and disadvantages. Epidemiological enquiries have to balance between lack of detailed measures and access to large numbers of participants for robust results. Lengthy clinical interviews are rarely a practical possibility. In addition, the use of a simple global measure of severity can have advantages over detailed interviews,^34^ particularly if it comes to an uncertain, subjective and multi-faceted construct like withdrawal. Assessment of withdrawal, similar to pain and low mood, is subjective. The advantage of self-report is that within-person changes over time are rated on the same subjective scale,^25^ although between-person comparisons may suffer in precision.

## Declarations

**Ethics approval and consent to participate:** Under Dutch law, medical ethical approval is not required for analysis of anonymous routine quality assessment data. On the form, patients are given information about the purpose of the questionnaire and were also informed that if they wished to consent to participation in anonymised research, they could send back the completed anonymous questionnaire. No identifiers were collected and no reminders were sent.

**Consent for publication:** Not applicable.

**Availability of data and materials:** Not applicable.
